# A New Method for Compression Testing of Reinforced Polymers

**DOI:** 10.3390/polym16213071

**Published:** 2024-10-31

**Authors:** Ciprian Ionuț Morăraș, Dorin Husaru, Viorel Goanță, Paul Doru Bârsănescu, Fabian Cezar Lupu, Corneliu Munteanu, Nicanor Cimpoesu, Elena Roxana Cosau

**Affiliations:** 1 Mechanical Engineering, Mechatronics and Robotics Department, Mechanical Engineering Faculty, “Gheorghe Asachi” Technical University of Iasi, 700050 Iasi, Romania; ciprian-ionut.moraras@academic.tuiasi.ro (C.I.M.); viorel.goanta@academic.tuiasi.ro (V.G.); paul-doru.barsanescu@academic.tuiasi.ro (P.D.B.); fabian-cezar.lupu@academic.tuiasi.ro (F.C.L.); 2 Fluid Mechanics, Fluid Machines and Fluid Power Systems Department, Machine Manufacturing and Industrial Management Faculty, “Gheorghe Asachi” Technical University of Iasi, 700050 Iasi, Romania; dorin-emil.husaru@academic.tuiasi.ro; 3 Technical Sciences Academy of Romania, 26 Dacia Blvd., 030167 Bucharest, Romania; 4 Department of Materials Science, Faculty of Materials Science and Engineering, “Gheorghe Asachi” Technical University of Iasi, 41 Dimitrie Mangeron Blvd., 700050 Iasi, Romania; nicanor.cimpoesu@academic.tuiasi.ro

**Keywords:** composite material, GFRP, compression test, Poisson’s ratio, FEA, force eccentricity, SEM analysis

## Abstract

Compressive testing of specimens taken from relatively thin composite plates is difficult, especially due to the occurrence of buckling. To prevent buckling, the central portion of the specimens used for the compression test has smaller dimensions, and the specimens can be guided along their entire length. For these reasons, optical methods, such as digital image correlation (DIC), cannot be used for the compression test and strain rosettes cannot be glued onto the samples to determine Poisson’s ratio. In this study, compression tests of a glass fiber-reinforced polymer (GFRP) were conducted using both the ASTM D695 (Boeing version) and a newly proposed method. The new method involves using special specimens that allow T-type rosettes to be bonded to determine Poisson’s ratio, whose value of 0.14 was thus determined. SEM images of the failure surfaces were presented and interpreted. A finite element analysis (FEA) of the specimens tested in compression is also presented. The first analyzed case considers the homogeneous and orthotropic composite, loaded with a uniformly distributed force. The normal stress in the central section of the specimen, determined with FEA, has an error of 6.52% compared to that determined experimentally. Additionally, the strain in the center of the strain gauge, determined with FEA, has an error of 4.76% compared to the measured one. In the second case studied with FEA, the sample is loaded with a quasi-concentrated force, which can move in the direction of the symmetry axes of the cross-section, to study the effect of the eccentricity of the compression force on the state of stress. It was shown that the eccentricity of the force has a great influence: the stress distribution in the section of the specimen becomes strongly non-uniform. For a force eccentricity of 0.4 mm in the direction of the OX axis, the minimum stress decreases by 53.7%, and the maximum stress increases by 55.4%. In order to analyze the influence of some manufacturing defects, two other cases were analyzed by FEA, in which it was assumed that the thicknesses of the outer resin layers were modified, making them asymmetrical. For this final FEA, the specimen was considered to be composed of laminates. These results demonstrate the special attention that must be paid to the centric application of force in compression testing.

## 1. Introduction

The actual way in which a fiber-reinforced composite material—fabric or unidirectional—is capable of taking external loads should still be explored. The variables that arise in this case are the type of matrix, the type and arrangement of the yarns/weave, the number of fabric layers and lay-up in the matrix, the external stress, and how it is applied in accordance with the type of stress introduced and its direction. For a pure tensile load along unidirectional fibers, the interaction between the fibers and the matrix is not complicated. External forces are mostly taken up by the fibers in the direction of the stress. In general, the deformation of glass-fiber-reinforced polymer (GFRP) fibers is low, with high strength. Thus, the matrix transfers the external loading to the fibers through shear stresses. The fibers, under tensile stress, remain straight as they are subjected to normal tensile stresses, with the matrix being used to support and protect the fibers, bearing a very small part of the external stress. In practice, the external load supported by the matrix will be in accordance with the small deformation to which it is subjected, this being imposed by the tensile deformation of the fibers. Things completely change if the same structure is subjected to compressive stress, the interaction between the fibers and the matrix being, in this case, much more complex. In this case, the matrix plays a fundamental role in supporting the fibers. Although the strength and stiffness of fibers can also be quite high under compressive stress to support the axial load, the interaction between the matrix and the fibers is much more complex [1]. In the scientific world, efforts are being made to understand the interactions between the matrix and the fibers, especially when the external stress induces normal compressive stresses. For a better understanding of the behavior of a composite material under compressive stresses, numerous experimental tests have to be performed, including observing the structure during load application by integrity monitoring methods, using DIC (digital image correlation), etc.

**Figure 1:**
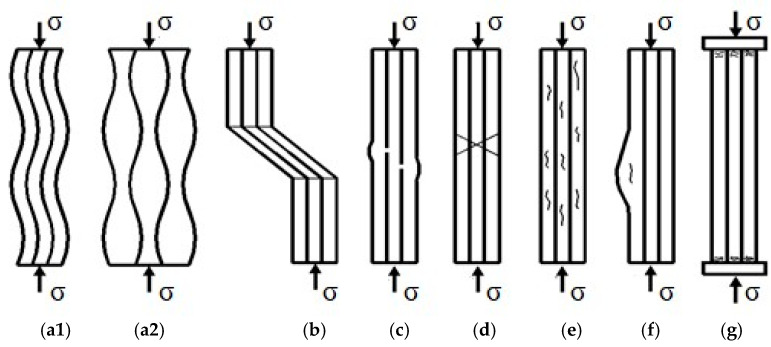
Compressive failure modes of fiber composites. (**a1**) Elastic micro buckling—in phase (shear microbuckling); (**a2**) Elastic microbuckling—out of phase (extensional microbuckling); (**b**) Fiber kinking; (**c**) Fiber crushing; (**d**) Shear band formation; (**e**) Matrix cracking; (**f**) Buckle delamination; (**g**) Contact damage [11,12,13].

Often, failure of components made of certain composite materials is caused by compressive stresses. This is caused by the fact that some of these materials can have compressive strengths as much as 30–40% lower than tensile strengths [2]. This is mainly due to the shape and arrangement of the fibers in the material, as well as their proportion in the base matrix. For polymer composites, identifying how they critically fail is not easy, especially when the stress is compounded, having both tensile and compressive normal stresses, as well as tangential stresses. In general, the failure of composite materials is catastrophic in nature, with no warnings of large deformation or failure to function beforehand [3]. In some research, several types of possible compressive failure have been observed, predicted, and reported: interfacial failure, Euler-type buckling, macrobuckling, elastic microbuckling of fibers, longitudinal splitting, interlaminar failure, specimen end crushing on contact with the test fixture, specimen shear failure, kink band formation, fiber failure, matrix failure, plastic microbuckling, etc. [4,5,6,7,8,9]. In view of all these possibilities of composite failure under compressive stress, a probabilistic distribution of the results obtained in this test is required [10]. Compressive failure is most likely caused by local instability of the fibers embedded in the matrix. Compressive stress failure is most often attributed to local instability of fibers embedded in the matrix. The local damage may start as a result of fiber waviness in the region with more resin, misalignment of the fibers with respect to the direction of normal stresses, free edge end effect, or poor bonding between the fibers and the matrix, as seen in Figure 1.

From previous studies, it appears that the fiber microbuckling failure mode is predominant for compressive testing of unidirectionally or multidirectionally reinforced fiber matrix composites [14]. This type of failure is locally initiated and propagates with increasing load, creating a narrow zone called the buckling band, which loses its structural integrity and collapses, as seen in Figure 1c. This effect is completely damaging in GFRP systems, even if they are bidirectionally or multidirectionally reinforced. It is true that in a bidirectional reinforcement, the fibers perpendicular to the stress direction can take up part of the deformations in that reinforcement direction, and the buckling phenomenon is reduced.

Due to the importance of determining the compressive stress behavior of fiber-reinforced composites, much research has been done in this area, and standards have been developed exactly for this purpose. ASTM D 3410/D 3410M [15], is a method of compressive testing of composite materials in which the introduction of compressive force into the specimen is accomplished by shear stresses at the wedge gripping interfaces used. The method presented by the above-mentioned standard is applicable to unidirectionally reinforced composites, wet-tow placement, textile, short-fiber composites, or other similar shapes. In ASTM D 6641/D 6641M-09 [16], the compressive strength and stiffness characteristics of the composite material are determined using a device for combined load compression (CLC) testing. The materials compression-tested by this method must be symmetrical in shape as well as in the arrangement of the reinforcement layers. There are two methods of load transmission: method A requires the specimen to be reinforced at the ends, and method B uses tabs at the ends of the specimen. A first essential requirement of any compressive stress applied to composite materials is that the ends of the specimen do not crush on contact with the platens of the testing machine, which transmit the axial forces. Procedure A is applicable to low-orthotropic materials: short-fiber composites, woven fabrics, and reinforced continuous-fiber laminates having more than 50% axially oriented (0°) plies. In any case, care shall be taken that, when using gripping grips, they do not crush the specimen in the gripping area, as local stress concentrators may occur. Tabs should necessarily be placed on the ends of the specimen for materials with higher orthotropy, including unidirectional composite materials. In D 6641, the compressive force is introduced into the specimen by the combined effect of axial end loading and shear loading. Unidirectional composite materials (0° ply orientation), as well as multidirectionally laminated composites, fabric composites, chopped fiber composites, and similar materials, may be subjected to this type of test.

The test method proposed by standard D695-15 [17] leads to the determination of the mechanical properties of high-modulus composites, as well as un-reinforced and reinforced rigid plastics, when loaded in compression at relatively low and uniform strain rates. To be tested using this method, the composite material must have a Young’s modulus of up to 41,370 MPa. Loading of the specimen by this method is accomplished simply by axial end loading.

In this research, compression tests were performed on specimens made of GFRP composite material. Two types of tests were performed based on the devices presented in the following section. A set of four specimens was tested according to the modified ASTM D695 standard (Boeing method), in which the compressive strengths and Young’s modulus of elasticity were determined. To prevent buckling, the central portion of the specimens used for the compression test has small dimensions (5 to 12 mm long and 10 to 15 mm wide), and the specimens can be guided along their entire length. For these reasons, optical methods (such as DIC) cannot be used for the compression test, and T-type strain rosettes cannot be glued to the samples to determine the Poisson’s ratio. To remove these disadvantages, a new testing method has been proposed, using a tower-type specimen. This test specimen allows the installation of T-type rosettes and the use of optical methods (such as DIC). Thus, the Poisson’s ratio of the GFRP composite was determined for compression.

A new compressive testing method, which aimed to avoid buckling, was proposed of the compression testing of four GFRP specimens in the elastic range. The Poisson’s ratio was also determined using this method. The results acquired from the compressive stress testing, which complied with the modified ASTM D 695, were compared with finite element analysis (FEA) in three ways. In the first case, the GFRP material was considered homogeneous and orthotropic, with the loading being applied as a uniformly distributed force. In the second case, the loading was applied using a quasi-concentrated force, considering the influence of run-out for this purpose. The last FEA case was analyzed by considering the layered GFRP material.

Surface and sectional electron microscopy (SEM) analyses were also performed on the specimens loaded up to failure. The SEM analyses helped to identify the fiber failure mode in the central fracture area, the reinforcement directions, and the categorization of the fracture mode to a specific type according into standard D 3410/D 3410M-03.

The novelty of this work consists of the fact that both compressive strength and elastic characteristics have been determined through different compression tests, with the experimental values obtained also being used in the FEM. SEM analysis of the fracture surfaces revealed the characteristics and modes of failure under compressive stress.

## 2. Materials and Methods

### 2.1. Materials

For this study, an RT 500 bi-directional fiberglass-reinforced composite board and MGS LR 385 EPIKOTE epoxy resin were utilized. The characteristics of the resin are shown in Table 1 [18].

### 2.2. Sample Preparation

**Figure 2:**
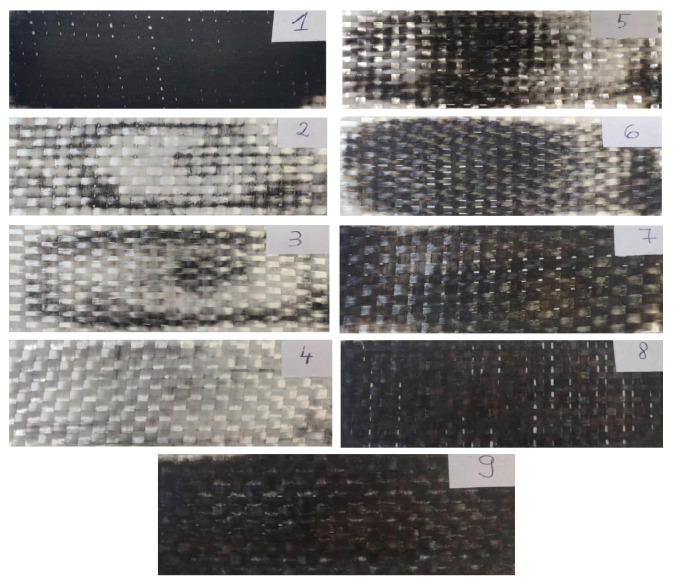
Fiberglass layers after the application of the complete combustion method (ASTM D 2584).

The GFRP plate was procured from the manufacturer “SC. Compozite Brasov SRL” (Romania) [10]. The manufacturing stages of the GFRP board were provided by the manufacturer and are described below. The composite plate was produced using the lay-up method. The first step consisted of selecting an unlaminated particleboard, which was cleaned, dried, and degreased with diluent. In order to avoid the sticking of the bottle fibers to the chipboard, 3 layers of liquid wax-based release agent were applied alternately. Each layer of release agent was left to dry for 20–30 min according to the manufacturer’s instructions, and finally, a finishing process was carried out. In the second step, the resin and hardener were weighed and then mixed together until homogenized. In the next step, each fiber layer was stacked and impregnated in the resin. A metal roller with circular channels was used to remove excess air between the fibers and the resin. The polymerization of all layers lasted for 24 h at a temperature of 20 °C. The last step included the detachment of the fiberglass composite board from the chipboard using plastic devices. The final result was a composite board composed of 9 layers with the fiber orientation at [0°/90°]. Figure 2 shows the fiberglass layers after the application of the complete combustion method, as indicated in ASTM D 2584. The GFRP sample (before combustion) and the fiberglass layers (after combustion) were weighed using an analytical balance. The fiberglass content (by weight) was 54.4%, and the epoxy resin content was 45.6%, as determined by this method.

Specimens were cut from the board using a diamond disk cutter, which limited heating in the cutting area and thus avoided edge effects (delaminations and fiber pull-outs). After the cutting process, the specimens were finished, resulting in a total of eight specimens taken in the transverse direction (TR): four specimens (100 × 16 mm) were used for the samples tested with the Boeing method (ASTM D 695), and another four specimens (130 × 25 mm) were used in a novel proposed compression test method.

### 2.3. Compression Tests

**Figure 3:**
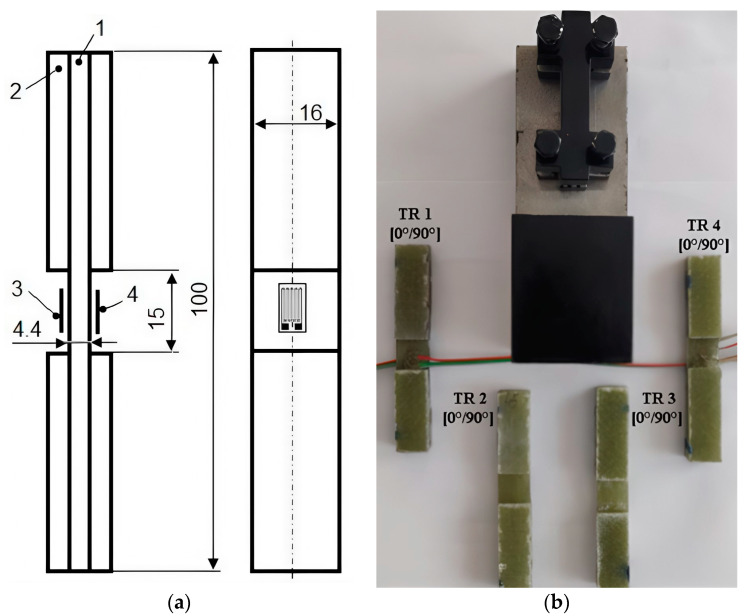
(**a**) GFRP specimen dimensions (in mm) according to ASTM D 695; (**b**) compression test device and test specimen set.

The mechanical behavior was analyzed during the compression test on a universal testing machine, type INSTRON 8801, which develops a maximum force of 100 kN. The tests were performed at room temperature, and the test regime was determined by a strain growth rate of 0.5 mm/min for each specimen. Due to the low speed of displacement during the tests, it was possible to observe the deformations occurring, thus obtaining a good accuracy in drawing the characteristic curves for specimens TR 1, 2, and 3. The compression tests were performed according to the modified ASTM D 695 standard (Boeing method) for specimens with [0°/90°] fiber orientation. The four specimens tested were cut in the transverse direction (TR) from the initial plate. In order to obtain the Young’s modulus, two unidirectional strain gauges of type EA-13-240LZ-120, manufactured by Micro-measurements, with a resistance of R = 120 Ω ± 0.3% and a mark factor of k_G_ = 2.095 ± 0.5%, were bonded to the TR 4 specimen. Under these conditions, the testing took place in the elastic range, both tensile marks bonded in the longitudinal direction measured the specific longitudinal strain, Ԑ_L_. In Figure 3a, the following notations were made: 1—specimen; 2—tabs; and 3 and 4—strain gauges bonded on both sides in the calibration area. Figure 3b shows the device and the specimens for the compression test, according to ASTM D 695 (Boeing method).

In the Boeing compression test method (modified ASTM D 695), the specimens are guided along their entire length to prevent buckling. For this reason, optical methods, such as digital image correlation (DIC), cannot be used. On the other hand, the above method uses specimens that are only 10 mm wide and 5–12mm long. This width is too small for the installation of strain gauge rosettes, which are necessary for determining the Poisson’s ratio. To address these disadvantages, a new test method has been proposed in the manuscript using a tower-type specimen. This test specimen allows the installation of strain gauge rosettes and the use of optical methods (DIC), thereby eliminating the disadvantages of the Boeing method. Testing the tower specimen until it breaks has the advantage of using a large volume of material. It is known that tests on large-volume specimens yield lower breaking strengths due to the accumulation of a greater number of defects in the material. In the tower specimen presented in the manuscript, the volume of the tested composite is approximately 50 times greater than that of the Boeing specimen.

**Figure 4:**
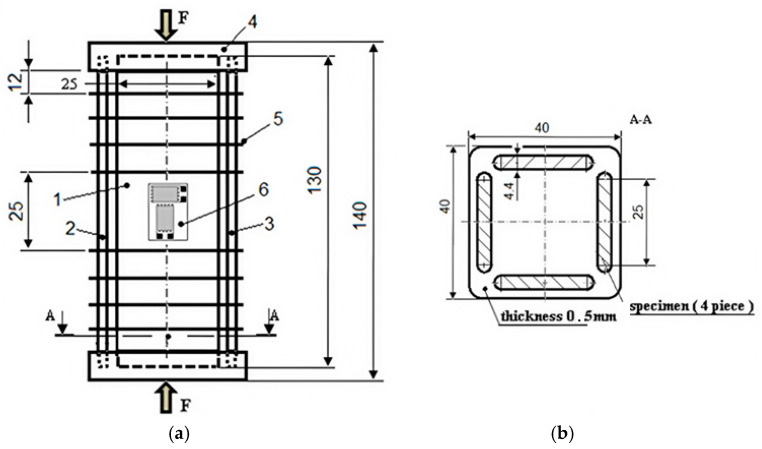
(**a**) Specimen for Poisson’s ratio determination (back GFRP sample not shown): 1—front sample; 2—left sample; 3—right sample; 4—Al alloy shell; 5—Al-sheet spacer; 6—T-type T-strain gauge; (**b**) Section A-A.

To determine the Poisson’s ratio of the studied GFRP, another compression test was performed using T-type strain gauges. These rosettes have a larger surface area than the usual (unidirectional) tensiometer marks and require a larger mounting space in the central area of the specimen. In order to meet these requirements, while also taking into account the need to avoid buckling, a special device has been developed in which four specimens of the same shape and size are tested simultaneously. The device groups four 130 × 25 mm specimens, cut from a 4.4 mm thick GFRP plate, placed along the sides of a square, with an aluminum alloy cover at each end and eight 0.5 mm thick aluminum sheet spacers at each end, as seen Figure 4.

**Figure 5:**
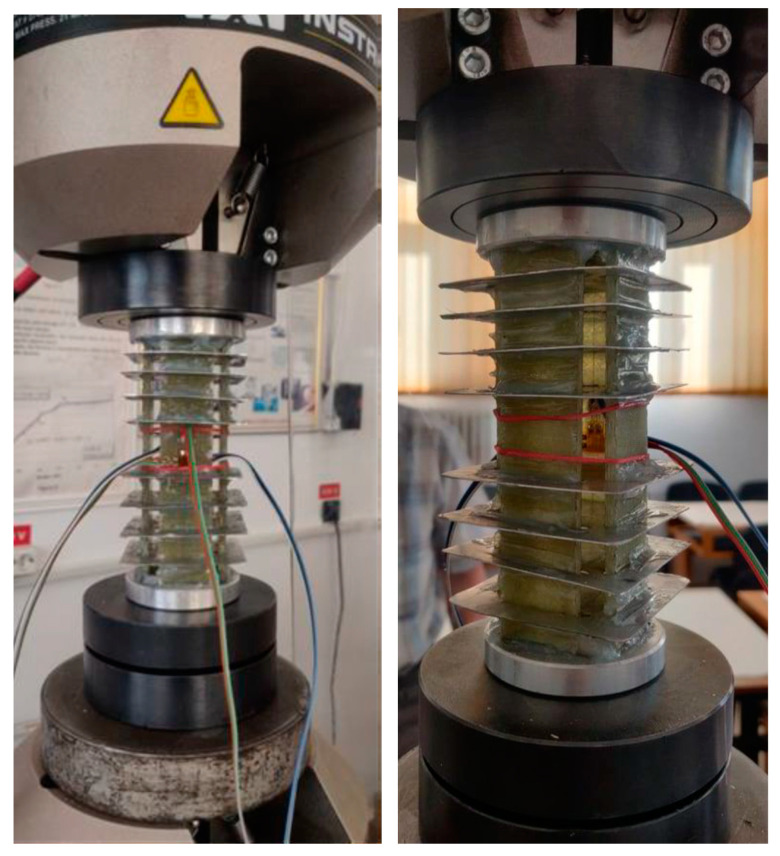
The compression device mounted on the INSTRON machine.

The aluminum alloy caps are 10 mm thick. Channels 4.5 mm deep were made in the caps, arranged as in the spacer shown in Figure 4. The ends of the four samples were inserted into these channels. The samples were fixed to the caps and spacers with adhesive. Thus, by arranging the four specimens along the sides of a square, the moment of inertia of the cross-section of the specimen was increased, and the spacers were used to decrease the buckling length (the buckling length remains longer only in the central part of the specimens where the strain gauge is mounted). On both sides of the front sample, two strain gauges of type CEA-06-125WT-120, manufactured by Micro-Measurements, with resistance R = 120 Ω ± 0.35% and brand factor k_G_ = 2.025 ± 0.5%, were glued to both faces. The four rosette grids were connected to a Vishay P3 tensiometer bridge (quarter-bridge mounted). The test specimen was placed between the platens of an Instron 8801 (Figure 5) testing machine, interspersed with a hemispherical hinge, to accommodate any deviations from parallelism between the upper and lower surfaces of the two covers. Loading of the specimen was conducted in the elastic range at a speed of 0.3 mm/min, up to a force of 8.2 kN for a single specimen. Force–displacement diagrams of the machine traverse and output signals from the four grids of the tensile rosettes were recorded during the test.

The specimen shown in Figure 4 also allows the use of high-performance optical methods for monitoring the strain and stress state of GFRP specimens, such as digital image correlation (DIC). The other standardized methods for compression testing (ASTM D 695-15, D 3410/3410M-03, D 6641/6641M-09) do not allow the use of DIC for design reasons.

### 2.4. Electron Microscopy Analyses

To highlight the morphology of the experimental specimens, an SEM microscope was used to analyze the mode of fracture in the area of interest of the GFRP composite material. The surface and cross-sectional fracture areas of the experimental materials, TR1, 2, and 3 were studied. To improve electrical conductivity, a Luxor Au SEM COATER Luxor Au—CT-2201-0144 was used, which realized a 7 nm gold layer on the surface and in the cross-section. A TESCAN Vega SEM microscope (Brno, Czech Republic) was used with the following parameters [19]: secondary electron (SE) detector, electron gun supply of 30 kV, high vacuum, and working distance of 15.5 mm.

## 3. Results and Discussion

### 3.1. Mechanical Properties of GFRP Under Compression Test at [0°/90°]

**Figure 6:**
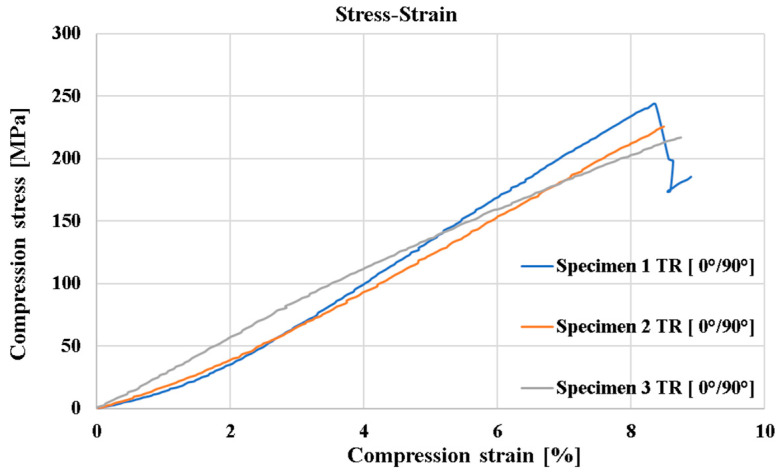
The stress–strain curves in the case of compression tests, analyzed until the samples’ breaking point.

Figure 6 shows the compressive behavior of GFRP-RT 500 specimens with [0°/90°] fiber orientation for the first compression test following the modified ASTM D695 standard (Boeing method). In the case of the three curves, it can be observed that the highest stress occurs at specimen 1, having the maximum value of σ_r_ = 243.23 MPa. By analyzing the appearance of the three plots, it can be observed that a sharp drop occurs at specimen 1 at a stress of about 240 MPa, which indicates a sudden breakage of a larger number of fibers. Similar diagrams have been obtained by other researchers [20].

**Figure 7:**
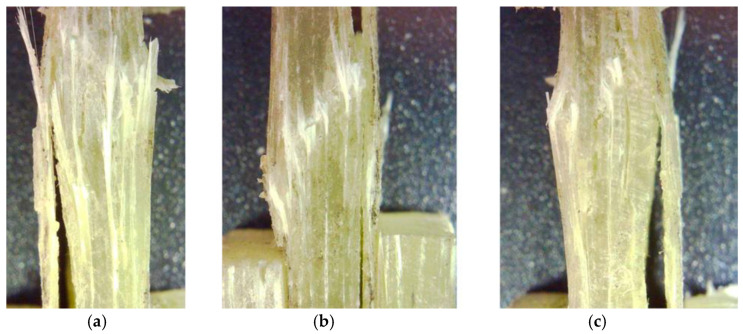
Failure types and failure modes for specimen TR of the plate [0°/90°]: (**a**) Specimen 1; (**b**) Specimen 2; (**c**) Specimen 3.

In the transverse cutting direction, noted as TR in the plate with fiber orientation [0°/90°], the types of breakage and failure modes were analyzed. These aspects complied with the typical failure codes/modes of the ASTD 3410/D 3410M—03. For specimens 1 and 3, the code BGM (brooming gage middle) wass used, and for specimen 2, the code was HAT (through-thickness at tab top), as shown in Figure 7.

In the following, a statistical analysis is presented for all sample sets from the plate with fiber orientation [0°/90°], as shown in Table 2.

**Figure 8:**
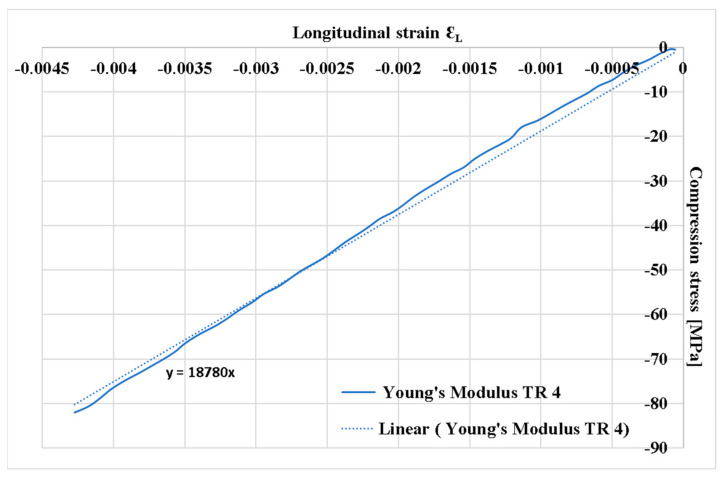
Stress variation with longitudinal strain: Approximation lines for the longitudinal elastic modulus at [0°/90°].

Figure 8 presents the normal stress (σ)/specific longitudinal strain (ε_L_) coordinate plots of the longitudinal modulus of elasticity, E (Young’s modulus), for the plate specimens TR4 with orientation [0°/90°]. Young’s modulus was determined as the slope of the approximation line of the plotted normal stress (σ)/specific longitudinal strain (ε_L_) coordinate plot through the points determined from the Instron 8801 force values and specific longitudinal strain records. Points located at low force values (influenced by the initial clamping processes in the machine’s bores) and points located at high force values, for which no strain recordings were made with the P3 bridge, were removed from the graph, as detachments of the tensiometer rosette and distortions of the strain recordings occurred.

**Figure 9:**
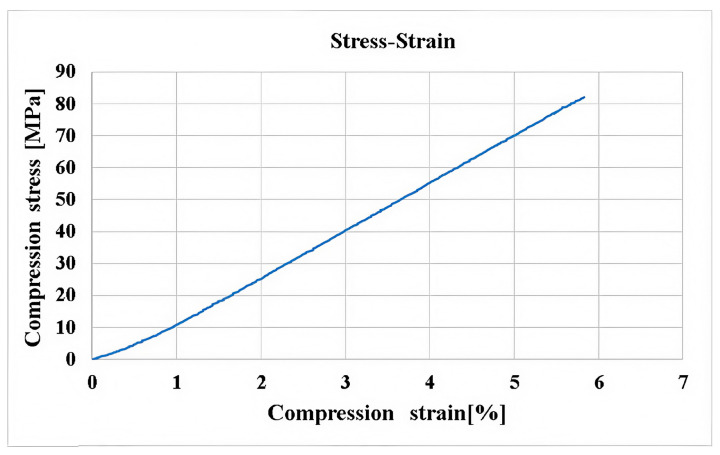
The stress–strain curve from compression tests at [0°/90°].

Figure 9 shows the stress–strain curve for a specimen subjected to compression, which represents the second test method in this research. The stress was within the elastic range; the curve is linear in appearance.

Since the output signals from the two longitudinally oriented grids satisfy the condition for maximum force,

$$\frac{\left|\varepsilon_{LF}-\varepsilon_{LB}\right|}{\left|\varepsilon_{LF}+\varepsilon_{LB}\right|}\cdot 100=\frac{6991-6027}{6991+6027}\approx 7.4\%<10\%$$

and according to ASTM D 3410/3410M-03, buckling does not occur in the samples.

The Poisson coefficient was calculated using the relation

$$\nu =\left|\frac{{∆\varepsilon }_{T}}{∆\varepsilon_{L}}\right|$$

**Figure 10:**
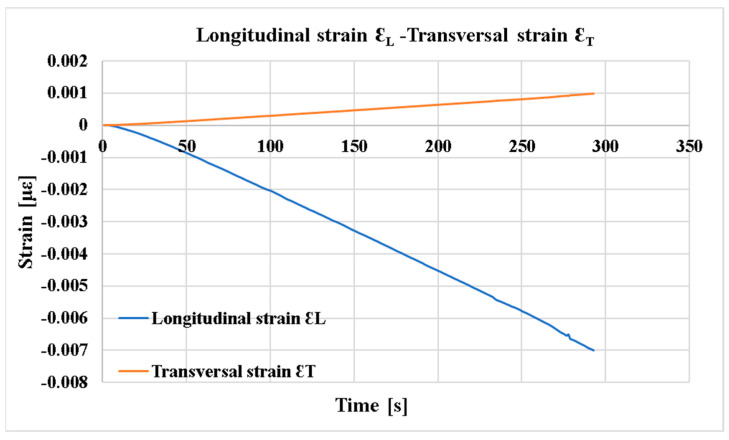
The Longitudinal strain (εL)–Transversal strain (εT) curve from compression tests at [0°/90°].

Figure 10 shows the output signals from the two rosette grids bonded to the front surface of the sample, recorded for the duration of the test.

**Figure 11:**
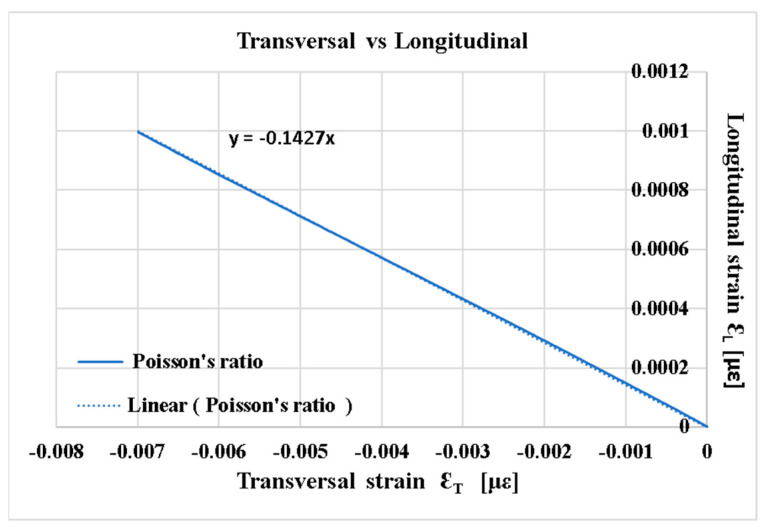
Longitudinal strain–transversal strain curve at [0°/90°].

Figure 11 shows the output signal of the transverse strain gauge versus the output signal of the longitudinal strain gauge.

The slope of this line, taken in absolute value, is the Poisson’s ratio. For the studied GFRP, the Poisson’s ratio ν = 0.1427 was obtained in compression. For the tensile test of cross-cut specimens from the same GFRP, ν = 0.1 was obtained [21].

### 3.2. SEM Surface at [0°/90°]

**Figure 12:**
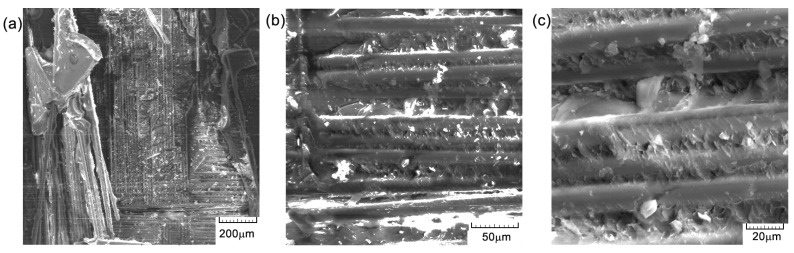
SEM images of the surface material—GFRP-specimen TR1: (**a**) 200×; (**b**) 500×; and (**c**) 1000×.

**Figure 13:**
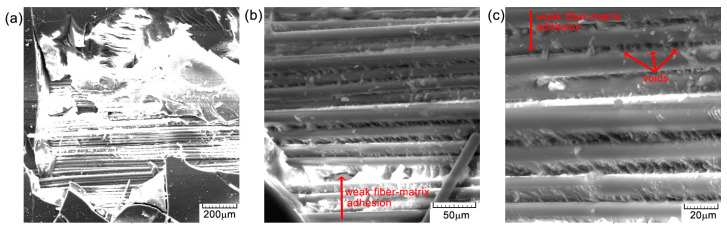
SEM images of the surface material—GFRP-specimen TR2: (**a**) 200×; (**b**) 500×; and (**c**) 1000×.

**Figure 14:**
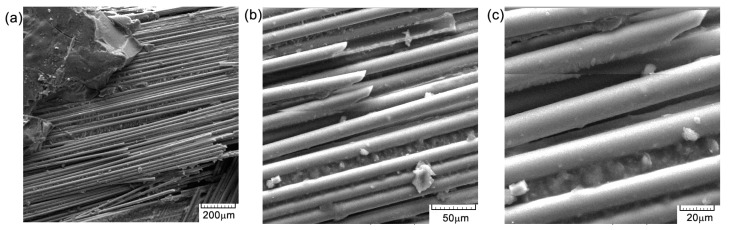
SEM images of the surface material—GFRP-specimen TR3: (**a**) 200×; (**b**) 500×; and (**c**) 1000×.

Figure 12, Figure 13 and Figure 14 illustrate several characteristics of the specimens under investigation that were subjected to compression tests at different levels of magnification. In Figure 13b,c and Figure 14, visual examination of the sample surface morphology reveals a uniform alignment of fibers within the matrix, with the presence of small voids at the fiber–matrix interfaces caused by the manufacturing and solidification processes of the epoxy resin. The adhesion of the fibers to the resin is thus deficient and can lead to decreased mechanical properties. The matrix exhibits attachment to the fibers, with very few cases of separation, as seen in Figure 13a and Figure 14a. The presence of microcracks (Figure 13a) or voids (Figure 13c), together with areas of weaker adhesion of the fiber matrix (Figure 13c), contributes to the initiation of composite damage and premature failure. The presence of these defects may also be due to the process of obtaining the composite material, particularly at the resin introduction stage. The composition of the material results in the formation of a robust substance that exhibits enhanced load-bearing capabilities [22], thus indicating a significant degree of impact resistance. The observed surface orientation of the tested fibers ranged from [0°/90°], with a notable failure occurring at 45 degrees (Figure 14b), indicating ductile behavior.

**Figure 15:**
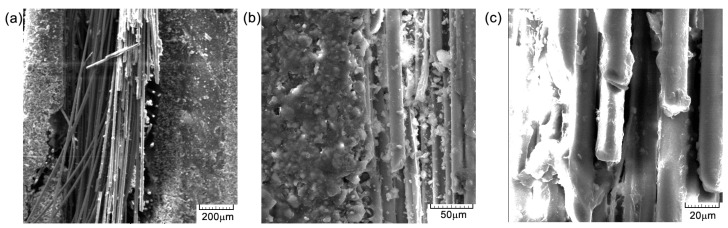
Cross-section SEM images of GFRP specimen TR1: (**a**) 200×; (**b**) 500×; and (**c**) 1000×.

**Figure 16:**
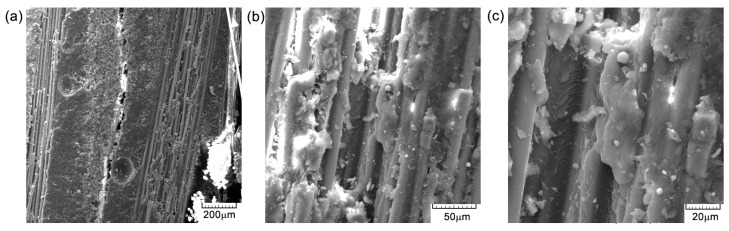
Cross-section SEM images of GFRP specimens TR2: (**a**) 200×; (**b**) 500×; and (**c**) 1000×.

**Figure 17:**
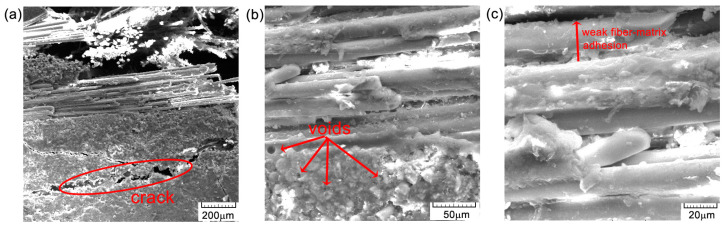
Cross-section SEM images of GFRP specimen TR3: (**a**) 200×; (**b**) 500×; and (**c**) 1000×.

The cross-sectional areaa of the specimens subjected to the compression test are noted in Figure 15, Figure 16 and Figure 17. From the compression tests and SEM analysis (Figure 12b and Figure 17b, respectively), a better surface adhesion of the reinforcing fibers to the resin compared to the cross-section is observed. The fiber separation at the contact with the matrix was seen to occur throughout an angular range of [0°/90°]. The longitudinal fractures seen in the base matrix, as shown in Figure 16a and Figure 17a, may be attributed to the presence of spherical voids inside the resin. The elements that induce fracture propagation and can lead to the formation of multiple cracks include porosity, fiber interfaces, and particle interactions.

### 3.3. Finite Element Analysis of GFRP

The finite element analysis of the specimen used in the modified ASTM D 695 compression test (Boeing method) was performed in ANSYS Academic 17.2 in two variants: The composite material was considered homogeneous and orthotropic, having the mechanical and elastic characteristics determined above; the composite material was considered to consist of layers (layers of fiberglass fabric alternating with layers of epoxy resin).

#### 3.3.1. Homogeneous and Orthotropic GFRP Analysis

Case 1. Loading with uniformly distributed force

**Figure 18:**
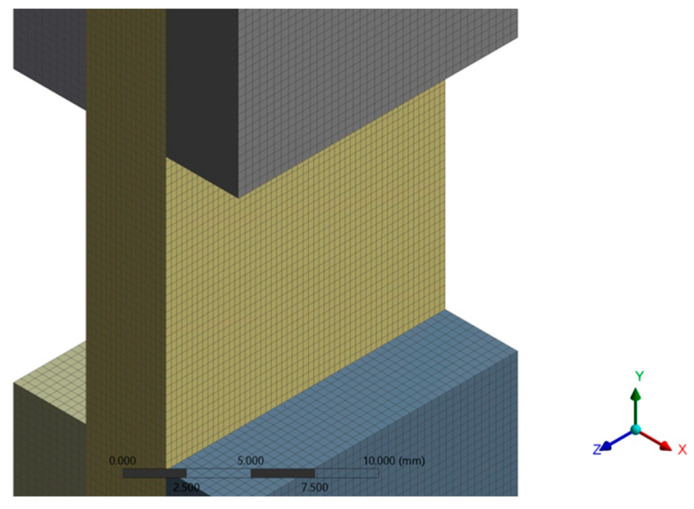
Mesh (central part of sample).

The discretization network consists of prismatic elements with a maximum size of 0.4 mm. It was obtained using a size function of uniform type, slow transition, and medium smoothing. The discretization network has 1,639,827 nodes and 379,056 finite elements (Figure 18).

**Figure 19:**
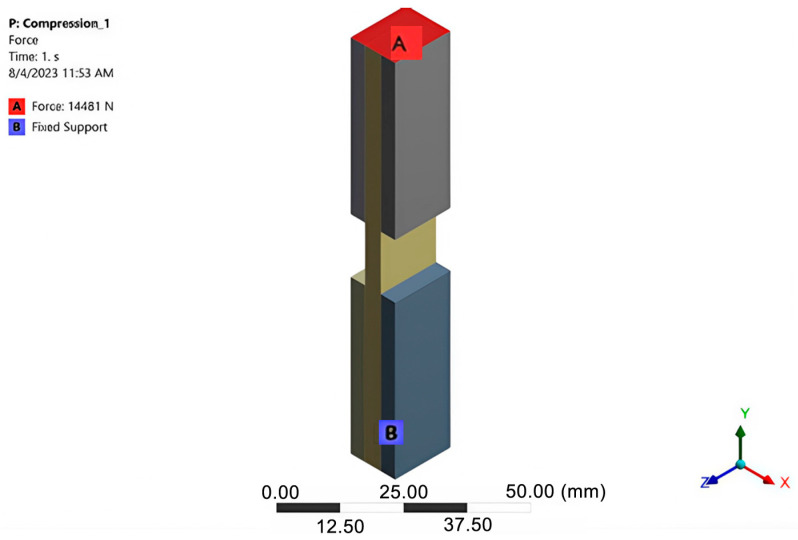
Fixed support and force applied to the specimen.

From a qualitative point of view, it is an excellent network, according to the quality parameters (orthogonal Quality, skewness, and aspect ratio). The loading was performed by applying a force distributed over the entire upper surface of the specimen, with a value of 14,481 N in the negative direction of the *Y*-axis. The specimen was considered embedded on the lower surface (Figure 19).

**Figure 20:**
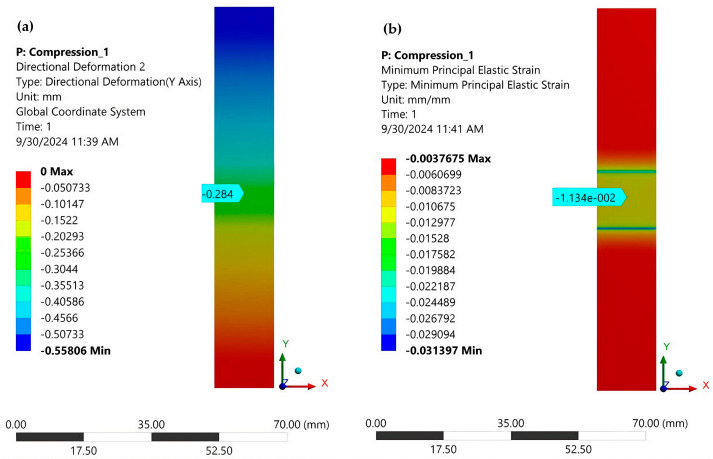
(**a**) The total displacement of the specimen in the *Y*-axis direction (without tabs); (**b**) specific strain distribution on the specimen with tabs removed.

Figure 20a shows the total displacement of the specimen (without tabs) in the *Y*-axis direction, and Figure 20b shows the strain ε_y_. The arrow indicates the specific strain from the center of the specimen, as well as from the center of the strain gauge glued to the specimen. The two blue-green colored bands mark the jump that occurs when switching from the part with tabs to the central side of the specimen. A similar jump occurs in the stress distribution. This represents a normal distribution in the area of stress concentrators. To decrease the maximum stress in this area, it is recommended to shear the tabs, thus achieving as smooth a transition as possible between the tabbed and central areas of the specimen.

**Figure 21:**
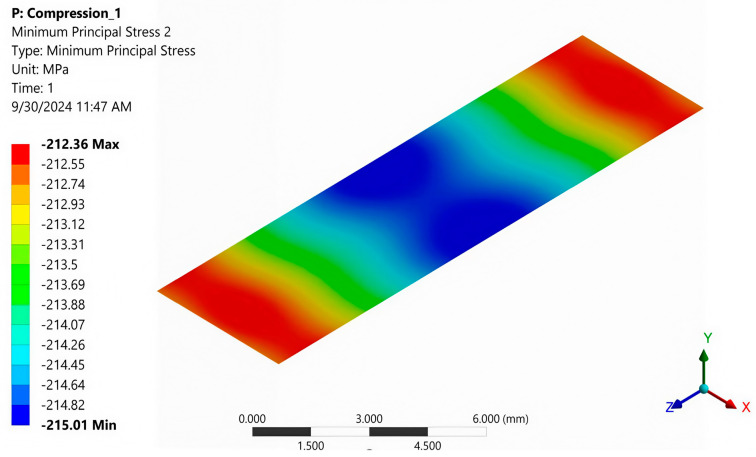
Normal stresses σ_y_ in the cross-section in the middle of the specimen (loaded with a uniformly distributed force).

**Figure 22:**
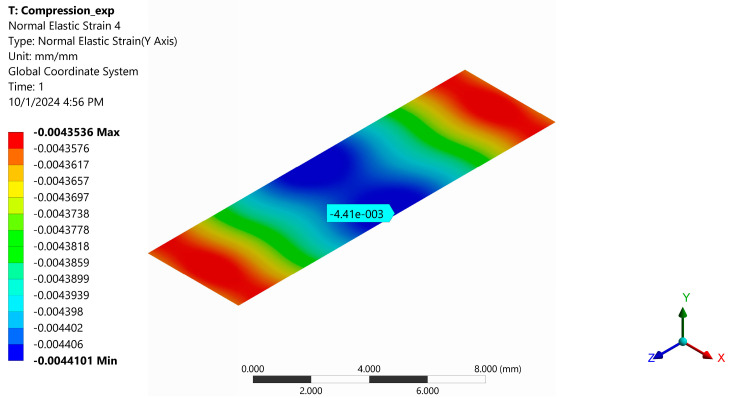
Normal elastic strain in the cross-section in the middle of the specimen (normal stress is 80 MPa). The strain in the middle of the strain gauge is indicated.

Figure 21 shows the normal stresses σ_y_ in the cross-section at the mid-section of the specimen when it is loaded with a uniformly distributed force. It can be seen that these stresses are practically constant over this cross-section, with the minimum stress being −215.01 MPa and the maximum being −212.36 MPa. Figure 22 depicts the strain distribution in the central section of the sample at a stress of 80 MPa, with the strain gauge center’s value indicated.

An average compressive strength of 228.62 MPa was experimentally determined (Table 1). Under the same loading conditions, a stress of 212.36 MPa was found by FEA (Figure 21). The relative error between the above two stresses is 7.11%. For a stress of 80 MPa, a strain of −0.0042 (Figure 8) was measured with a strain gauge, and −0.0044 was determined using FEA (Figure 22). The strain value provided by FEA has a relative error of 4.5% compared to the measured value. These errors are acceptable for the engineering field and validate the correctness of the FEA.

Case 2: Loading with quasi-concentrated force. The influence of load eccentricity was simulated using FEA. A quasi-concentrated force (distributed on the surface of a square with a side length of only 0.4 mm) of 14,481 N was applied to the specimen. The discretization network has 2,471,873 nodes and 581,160 finite elements. The other network parameterizations are identical to those in case 1.

**Figure 23:**
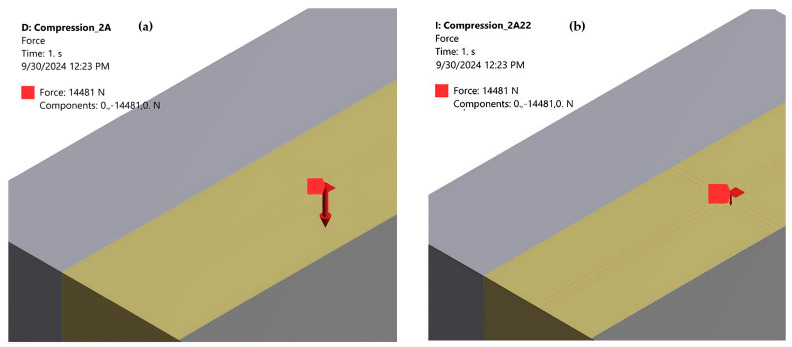
(**a**) Loading the specimen with quasi-concentrated force; (**b**) loading the specimen with an eccentric force displaced by 0.4 mm in the OZ direction.

Initially, the center of the square coincides with the center of gravity of the cross-section (Figure 23a). In this case, the discretization network is modified due to the fact that the upper surface is divided into several finite elements. For both uniformly distributed force loading and quasi-concentrated force loading, the same stress state is obtained in the cross-section at the middle of the specimen, as shown in Figure 21 and Figure 22 (small differences occur only at the second decimal place). However, in the area of application of the two forces, there are significant changes in the stress state, deformations, and displacements, which are shown here. This confirms Saint-Venant’s principle as used in the Theory of Elasticity and Strength of Materials.

Next, the center of the square in which the force is applied was displaced in the direction of the OZ and OX axes, respectively (displacements of 0.2 mm and 0.4 mm, respectively, as shown in Figure 23b). The influence of these eccentricities on the stress, strain, and displacement states of the specimen was studied.

**Figure 24:**
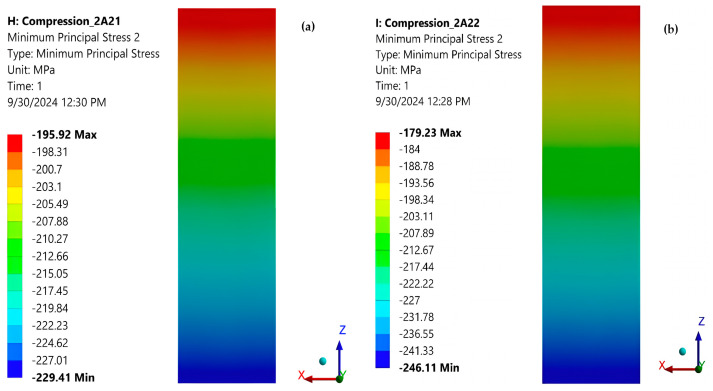
(**a**) Minimum principal stresses σ_y_ in the cross-section at the middle of the specimen (loaded with a quasi-concentrated force with an eccentricity of 0.2 mm, in the direction of the OZ axis); (**b**) Minimum principal stresses σ_y_ in the cross-section at the middle of the specimen (loading with a quasi-concentrated force with an eccentricity of 0.4 mm in the direction of the OZ axis).

**Figure 25:**
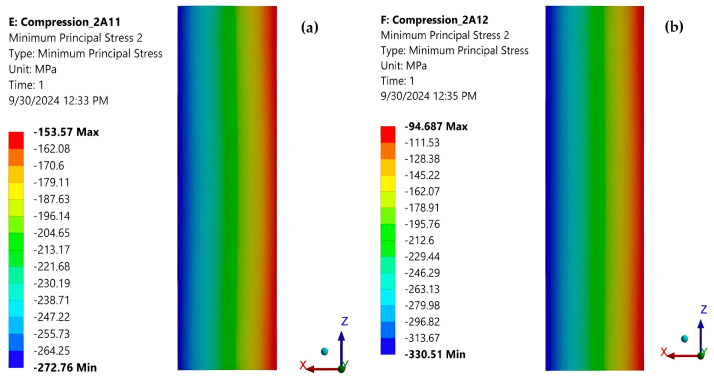
(**a**) Minimum principal stresses σ_y_ in the cross-section at the middle of the specimen (loaded with a quasi-concentrated force with an eccentricity of 0.2 mm in the direction of the OX axis); (**b**) Minimum principal stresses σ_y_ in the cross-section at the middle of the specimen (loading with a quasi-concentrated force with an eccentricity of 0.4 mm in the direction of the OX axis).

Figure 24a and Figure 24b show the minimum principal stresses σ_y_ in the mid-section of the specimen for force eccentricities of 0.2 mm and 0.4 mm, respectively, in the OZ direction. It can be seen that bending of the specimen also occurs due to the eccentrically applied force. When the force moves in the direction of the OZ axis, the neutral axis is parallel to OX, and when it moves in the direction of OX, the neutral axis is parallel to OZ (Figure 24a,b; Figure 25a,b).

Table 3 shows the extreme values of the minimum principal normal stresses σ_y_ in the cross-section at the center of the specimen for the above loading cases. It can be seen that even relatively small eccentricities significantly change the stress state in the specimen midsection, especially if the force is traveling along the OX axis (across the specimen thickness). For this reason, special measures are necessary to decrease the eccentricity in the compression of composites.

Using the ASTM D 695 method (Boeing variant), the average compressive strength of the composite was determined to be 228.6 MPa (Table 1). On the other hand, the average normal stress of about 213.7 MPa was found by FEA under the same loading condition (Table 2). The relative error between the two above stresses is 6.52%. For the stress of 80 MPa, a strain of −0.0042 (Figure 8) was measured with a strain gauge, and a strain of −0.0044 was determined with FEA (Figure 22). The value of strain provided by FEA has a relative error of 4.76% compared to the measured value. These errors are acceptable for the engineering field and validate the correctness of the FEA. In Table 2, it can be seen that the eccentricity of the force has a significant influence: The stress distribution in the section of the specimen becomes strongly non-uniform. For an eccentricity of 0.4 mm in the direction of the OZ axis, the minimum stress decreases from −215.01 MPa to −246.11 MPa (14.46% relative error), and the maximum stress increases from −212.36 MPa to −179.23 MPa (15.6% relative error). For an eccentricity of 0.4 mm in the direction of the OX axis, the minimum stress decreases from −215.01 MPa to −330.51 MPa (53.7% relative error), and the maximum stress increases from −212.36 MPa to −94.69 MPa (55.4% relative error). These results demonstrate the special attention that must be paid to the centric application of the force in the com-pression test.

#### 3.3.2. Analysis of GFRP as a Layered Material

**Figure 26:**
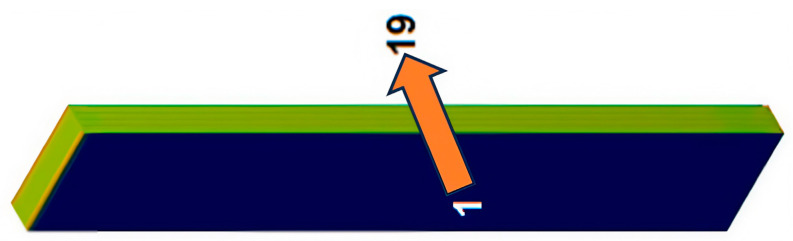
Numbering of resin and fiberglass layers.

The analyzed material has nine layers of [0°/90°] fiberglass fabric and ten layers of resin (distributed inter-laminarly and at the composite surface). The numbering of the layers was done in ascending order, starting from the face shown in Figure 26. Layer 10 is fiberglass cloth and contains the plane of symmetry of the specimen. The thickness of the plate from which the specimens were cut is 4.4 mm.

The thickness of the fiberglass fabric layers is 0.4 mm. To begin with, the thickness of all resin layers was considered equal to 0.08 mm, and the layers are arranged symmetrically (for case A). In order to analyze the influence of certain manufacturing defects, two other cases were analyzed in which it was assumed that the composite board resulted in the same thickness, but the thicknesses of the outer resin layers were modified as shown in Table 4, and the thicknesses of the inner resin layers were not affected.

**Figure 27:**
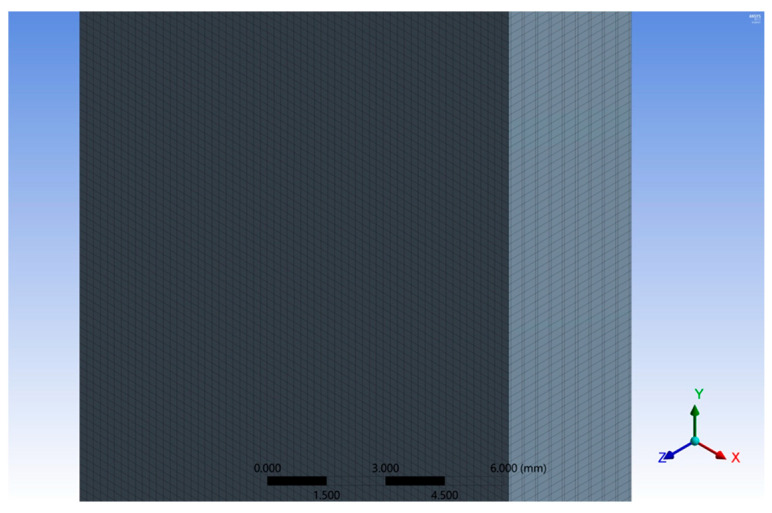
The discretization network of the stratified test specimen.

The discretization network consists of prismatic elements with a maximum size of 0.4 mm, which is the maximum size of the reinforcement layer shown in Figure 27. The discretization network is quantitatively characterized by a total of 505,260 nodes and 471,200 elements, and qualitatively, it is an excellent network according to the quality parameters (orthogonal quality, skewness, and aspect ratio).

For these cases, only the specimen without tabs was used. The same force of 14,481 N was applied to its upper surface, oriented toward the negative side of the *Y*-axis (compression), and the specimen was embedded in the lower part.

**Figure 28:**
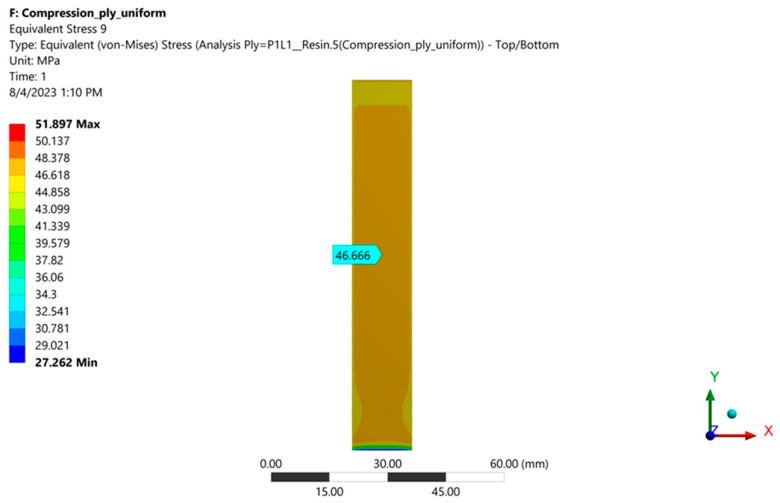
von Mises stresses in the middle of layer 9 (resin).

**Figure 29:**
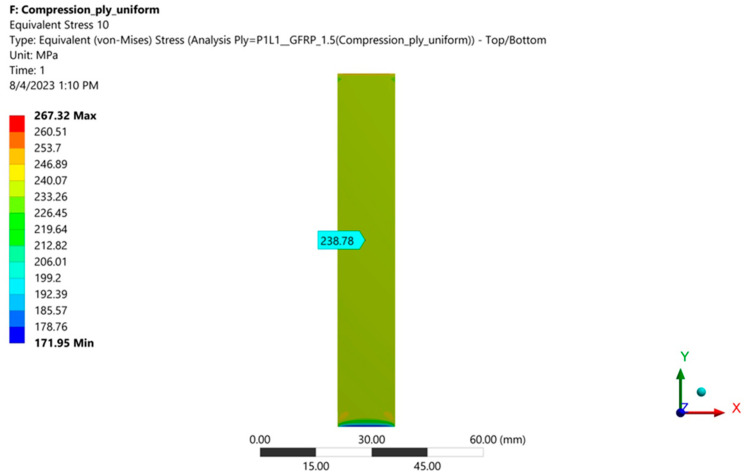
von Mises stresses in the middle of layer 10 (fiberglass fabric, which contains the plane of symmetry of the specimen).

Figure 28 and Figure 29 show the von Mises stresses in layers 9 (resin) and 10 (fiberglass fabric) for case A (layers arranged symmetrically).

Data on von Mises stresses in each layer and for each studied case are presented in Table 5.

It is observed that in case A, the symmetric layer loadings are the same. In cases B and C, the stresses increase in layers 1–10 as the outer layer 1 (resin) becomes thinner. In contrast, the stresses decrease in layers 11–19 as the outer layer 19 (resin) becomes thicker. The von Mises stresses in the most stressed fiberglass layer (4) increase from 266.66 MPa (case A) to 275.52 MPa (case C), i.e., by 3.3%.

## 4. Conclusions

1. The behavior of the GFRP composite material (54.4% fiberglass and 45.6% epoxy resin) subjected to compression was studied according to ASTM D695 using the modified Boeing method. The elastic and mechanical characteristics were determined, obtaining a Young’s modulus of E = 18,780 MPa, a compressive strength of 228.6 MPa, and a Poisson’s ratio of ʋ = 0.14.
2. SEM analyses were performed on the surface of the specimens broken by the Boeing method, as well as in the broken section. On the surface, SEM analyses showed uniform fiber morphology within the matrix. In the cross-section, microcracks specific to the compression test were observed.
3. The normal stress in the central section of the specimen determined with FEA has an error of 6.52% compared to that determined experimentally. The influence of the eccentricity of the force was also studied. A “concentrated” force was assumed to be uniform on the surface of a square with a side of 0.4 mm. This force was then displaced in the sectional plane along the OX and OZ axes, and the effect of force eccentricity was studied. It was thus demonstrated that small eccentricities significantly alter the stress state in the mid-section of the sample. The stress distribution becomes strongly non-uniform: for an eccentricity of the force of 0.4mm in the direction of the OX axis, the minimum stress decreases by 53.7%, and the maximum stress increases by 55.4%. For this reason, in the case of compression of composite materials, particular attention must be paid to minimizing the eccentricity of the resultant force.
4. In order to analyze the influence of certain manufacturing defects, two other cases were analyzed by FEA in which it was assumed that the composite board had the same thickness; the thicknesses of the inner resin layers were not affected, but the thicknesses of the outer resin layers were modified to become asymmetrical so that the sum of the thicknesses of the two outer resin layers remained constant. For this last FEA, the specimen is considered to be composed of laminates (nine fiberglass layers and ten resin layers). The two external resin layers were initially considered to have a thickness of 0.08 mm. In the case with outer layers of 0.04 mm and 0.12 mm, respectively, the von Mises stress increased by 3.3% for the most stressed layer (laminate no. 4).
5. From the results obtained with FEA, for the composite considered homogeneous and orthotropic, a maximum von Mises stress of 215 MPa was obtained, and for the material considered layered, a von Mises stress of 267 MPa was obtained. Experimentally, an average maximum normal stress of 228.62 MPa was obtained.

## Figures and Tables

**Table 1:** Characteristics of epoxy resin type EPIKOTE MGS LR 385.

Properties	Units	Value
Density	[g/cm^3^]	1.20
Viscosity	[mPa_x_s]	700–1050
Flexural strength	[N/mm^2^]	120–130
Modulus of elasticity	[kN/mm^2^]	3.3–3.6
Tensile strength	[N/mm^2^]	75–85
Compressive strength	[N/mm^2^]	120–140
Elongation of break	[%]	6–8
Impact strength	[KJ/m^2^]	45–60
Water absorption at 23 °C in 24 h	[%]	0.01

**Table 2:** The standard deviation for ultimate stress, σ_UTS_.

Sample No.	σ_UTS_ [MPa]	${\bar{\sigma }}_{UTS}$ [MPa]	Deviations from the Mean $\sigma_{UTS}-{\bar{\sigma }}_{r}$ [MPa]	${\left(\sigma_{r}-{\bar{\sigma }}_{r}\right)}^{2}$ [MPa]	Standard Deviation S [MPa]	Coefficient of Variation CV [%]	
1	243.23	228.62	14.61	213.45	13.30		5.81
2	225.49		−3.13	8.94			
3	217.15		−11.47	131.56			
Σ=	685.67			353.95			

**Table 3:** Extreme values of the minimum principal normal stresses σ_y_ in the cross-section from the middle of the specimen for different eccentricities.

Eccentricity e [mm]	Direction of Displacement Force	σ_y_ [MPa]		
		Minim	Maxim	Average
0	-	−215.01	−212.36	−213.68
0.2	OZ	−229.41	−195.92	-
0.4	OZ	−246.11	−179.23	-
0.2	OX	−272.76	−153.57	-
0.4	OX	−330.51	−94.687	-

**Table 4:** The thickness of the outer layers (resin) for the cases studied.

Case	Thickness of External Layers [mm]	
	Layer 1	Layer 19
A	0.08	0.08
B	0.06	0.1
C	0.04	0.12

**Table 5:** Equivalent von Mises stress in all layers for cases A, B, and C [MPa].

**Layer**	**1**	**2**	**3**	**4**	**5**	**6**	**7**	**8**	**9**	**10**
Case A	46.67	238.78	46.67	266.6	46.67	238.8	46.67	266.66	46.67	238.7
Case B	47.68	243.96	47.45	271.0	47.23	241.1	47.01	268.81	46.79	239.2
Case C	48.71	249.13	48.23	275.2	47.79	244.2	47.35	270.31	46.91	239.2
**Layer**	**11**	**12**	**13**	**14**	**15**	**16**	**17**	**18**	**19**	
Case A	46.67	266.66	46.67	238.78	46.67	266.66	46.67	238.78	46.67	
Case B	46.57	265.89	46.35	236.90	46.13	263.29	45.91	234.55	45.69	
Case C	46.47	265.10	46.03	235.01	45.59	259.89	45.15	230.31	44.71	

## Data Availability

The original contributions presented in the study are included in the article, further inquiries can be directed to the corresponding author.
